# Uranium capture from aqueous solution using palm-waste based activated carbon: sorption kinetics and equilibrium

**DOI:** 10.1007/s10661-024-12560-y

**Published:** 2024-04-04

**Authors:** W. M. Youssef, M. M. El-Maadawy, A. M. Masoud, I. G. Alhindawy, A. E. M. Hussein

**Affiliations:** Nuclear Materials Authority, Cairo, Egypt

**Keywords:** Uranium, Sorption, Aqueous solution, Palm and kinetic

## Abstract

Carbonaceous materials produced from agricultural waste (palm kernel shell) by pyrolysis can be a proper type of low-cost adsorbent for wide uses in radioactive effluent treatment. In this context, the as-produced bio-char (labeled as PBC) and its sub-driven sulfuric acid and zinc oxide activated carbons (labeled as PBC-SA, and PBC-Zn respectively) were employed as adsorbents for uranium sorption from aqueous solution. Various analytical techniques, including SEM (Scanning Electron Microscopy), EXD (X-ray Diffraction), BET (Brunauer–Emmett–Teller), FTIR (Fourier Transform Infrared Spectroscopy), and Zeta potential, provide insights into the material characteristics. Kinetic and isotherm investigations illuminated that the sorption process using the three sorbents is nicely fitted with Pseudo-second-order-kinetic and Langmuir isotherm models. The picked data display that the equilibrium time was 60 min, and the maximum sorption capacity was 9.89, 16.8, and 21.9 mg/g for PBC, PBC-SA, and PBC-Zn respectively, which reflects the highest affinity for zinc oxide, activated bio-char, among the three adsorbents, for uranium taking out from radioactive wastewater. Sorption thermodynamics declare that the sorption of U(VI) is an exothermic, spontaneous, and feasible process. About 92% of the uranium-loaded PBC-Zn sorbent was eluted using 1.0 M CH_3_COONa sodium ethanoate solution, and the sorbent demonstrated proper stability for 5 consecutive sorption/desorption cycles.

**Supplementary Information:**

The online version contains supplementary material available at 10.1007/s10661-024-12560-y.

## Introduction

Uranium is a radioactive heavy metal that has been released into the environment through natural and human processes (Akash et al., 2022). This dispersal has caused contamination of water sources and other environmental surfaces(Prasad et al., 2019). Even small amounts of uranium in water supplies pose a significant risk to the health of people, animals, and the environment due to its high toxicity, long half-life, and ability to form complexes with organic ligands (Bjørklund et al., 2020). However, nuclear energy is considered a superior energy source in the modern era, especially as the global population and energy needs rise, because of its lack of greenhouse gas emissions (Siqueira et al., 2019). Therefore, it is crucial to recover and reclaim U(VI) from polluted sites to advance the sustainable development of nuclear energy, protect the environment, and maintain human health.

The removal of U(VI) from liquid solutions has been accomplished using a variety of techniques, including membrane separation (Torkabad et al., 2017), co-precipitation (Dinis, & Fiúza, 2021), ion exchange (Naushad et al., 2015), electrocoagulation (Nariyan et al., 2018), solvent extraction (Ali et al., 2014), solid-phase extraction (Massoud et al., 2019; Morsy et al., 2019; Youssef, 2022), and sorption (Ali et al., 2019; El-Maadawy, 2019; Masoud et al., 2020; Youssef et al., 2022). Among these established methods, research has demonstrated that sorption is exceptionally effective in eliminating solutes using selective adsorbents. This approach is favored due to its cost-effectiveness, simplicity, rapid kinetics, broad applicability, and reduced potential for secondary contamination (Xiong et al., 2022).

Biosorption is a process where a biological entity produces a material that can accumulate the heavy metals in its cellular structure (Bayuo et al., 2020, 2022a, 2023a; Bayuo, 2021a). Biosorption is a rapid and reversible process in which the ions get bonded to the functional groups that are available on the surface of biomass like bio-char, marine alga, chitin, etc. (Bayuo et al. 2021, b; Pandiyarajan et al. 2023). Bio-char which is created from abundant animal waste and plant biomass boasts advantageous characteristics like strong permeability, great porosity, and a large surface area therefore it has been utilized in several environmental applications (Dong et al., 2022; Shen et al., 2023). By transforming agricultural solid waste such as palm biomass waste into a valuable product like an affordable adsorbent, a significant portion of it can be utilized and its economic value maximized while also contributing to waste reduction (Bayuo et al., 2023b; Bayuo, 2021b; Gozan et al., 2023; Mahlia et al., 2019).

Recently, there has been a growing focus on improving the efficiency of activated carbons with the help of various techniques aimed at enhancing their specific properties. Zinc chloride (ZnCl_2_) has gained a lot of interest as a potent chemical activation agent used to produce porous carbon compounds from different biomass sources. This is because ZnCl_2_ is a Lewis acid and can dehydrate biomass by removing only hydrogen and oxygen (Li et al., 2020). Additionally, the acidic treatment approach is the most researched among the employed techniques (Naji & Tye, 2022).

In this contribution, this study examines the effectiveness of bio-char derived from palm kernel shells and its sub-driven sulfuric acid and zinc chloride activated carbonaceous species to remove uranium from aqueous solution. The study examines the structural and morphological features of the three sorbents using SEM, EXD, N_2_ sorption–desorption isotherms, FTIR, and Zeta potential analysis. The impact of the main variables on uranium sorption from aqueous solution using the prepared three sorbents is investigated. The key parameters are optimized, and kinetics, isotherm, and thermodynamics of the uranium sorption process are analyzed to provide insights for sustainable water treatment.

## Methods

### Materials and analytical procedure

Reagent-grade sulfuric acid (H_2_SO_4_, ≥ 98%) and zinc chloride (ZnCl_2_, ≥ 99%) were sourced from Adwic, Egypt, and used as they were received. Analytical-grade uranyl nitrate hexahydrate (UO_2_ (NO_3_)_2_·6H_2_O) was purchased from Sigma-Aldrich USA and utilized to prepare a uranium stock solution at a concentration of 1000 ppm. Deionized water was employed to create various fresh solutions, each with a specific initial U(VI) concentration derived from the stock solution.

### Preparation of the modified carbon

Palm bio-char **(**labeled as **PBC**), which was produced from palm, was transferred by one-step chemical activation using 50% H_2_SO_4_ (labeled as **PBC-SA**), and 1:2 ZnCL_2_ (labeled as **PBC-Zn**). In each experiment, 50 g of palm was carbonized at 600 °C for 1 h. then the drying of palm was soaked in 50% sulfuric acid (Olivares-Marín et al., 2012), ZnCL_2_ ratio of 1:2 (Zhang et al. 2020)_._ The palm biomass was immersed in 150 mL of a modification solution, and gently agitated to ensure the acid penetrated evenly. The mixture was then heated to 80 °C for 1 h and left overnight at room temperature to facilitate proper wetting and impregnation of the precursor. Subsequently, the product underwent thorough washing with hot distilled water and was finally dried at 110 °C (Heidarinejad et al., 2020).

### Instruments

The morphology of sorbent beads was examined by scanning electron microscope (SEM) using QUANTA 200 with ≥ 10 kV accelerated voltage and Infrared spectrum (FTIR) for the sorbent beads was examined using the mid-infrared region from 4000 to 500 cm^−1^ using FT-IR spectrometer (Bomen, Hartman & Braun, and model MB-157, Canada) under ambient air condition using KBr as a diluent. A dynamic light scattering (DLS, Malvern-ZS, Ltd., UK, nano series) was employed for the zeta potential measurements. UV–vis spectrophotometer model SP-8001 UV-, Metretech Inc., version 1.02, 2000/10/01 was used for the determination of uranium concentration in the aqueous phase, as well as the constituents in the real sample.

### Equilibrium studies

Using the batch method, the sorption of uranium from an aqueous solution was investigated using (PBC), (PBC-SA), and (PBC-Zn) in a polypropylene tube. The study examined the effects of significant factors on U(VI) sorption process. To assure the attainment of equilibrium, a specific weight (*m*, g) of sorbent was stirred for 4 h at room temperature with an adequate volume (*V*, *L*) of an aqueous solution of uranium(VI) with an initial concentration of 50 ppm (*C*_*o*_, ppm). Following that, a solid/liquid separation was performed using a filter membrane with 1.2 µm. The remaining uranium concentration (*C*_*e*_, ppm) was determined spectrophotometrically using the Arsenazo III method at *λ* = 654 nm (Marczenko & Balcerzak, 2000) based on a calibration curve with a determination coefficient (*R*^2^) = 0.99 (see Figure S1). A pH adjustment was made to the solution using 0.5 M HCl and 0.5 M NaOH. The sorption experiments were carried out in triplicates and the mean value of ≤ 4% relative error was accepted.

Uranium (VI) sorption capacity (*q*_*e*_, mg g^−1^), sorption percent (%), and the distribution coefficient (*K*_*d*_) were evaluated using Eqs. 1, 2, and 3  respectively:

1 $${q}_{e}=\left({\text{C}}_{\text{o}}-{C}_{e}\right)\times \frac{V}{m}$$

2 $${\text{U}}({\text{VI}})\mathrm{ removal percent}=\frac{\left({C}_{o}-{C}_{e}\right)}{{C}_{o}}\times 100$$

3 $${K}_{d}=\frac{\left({C}_{o}-{C}_{e}\right)}{{C}_{e}}\times \frac{V}{m}$$

## Results and discussion

### Result of the modified carbon characterization

#### SEM and EDX analysis

**Fig. 1:**
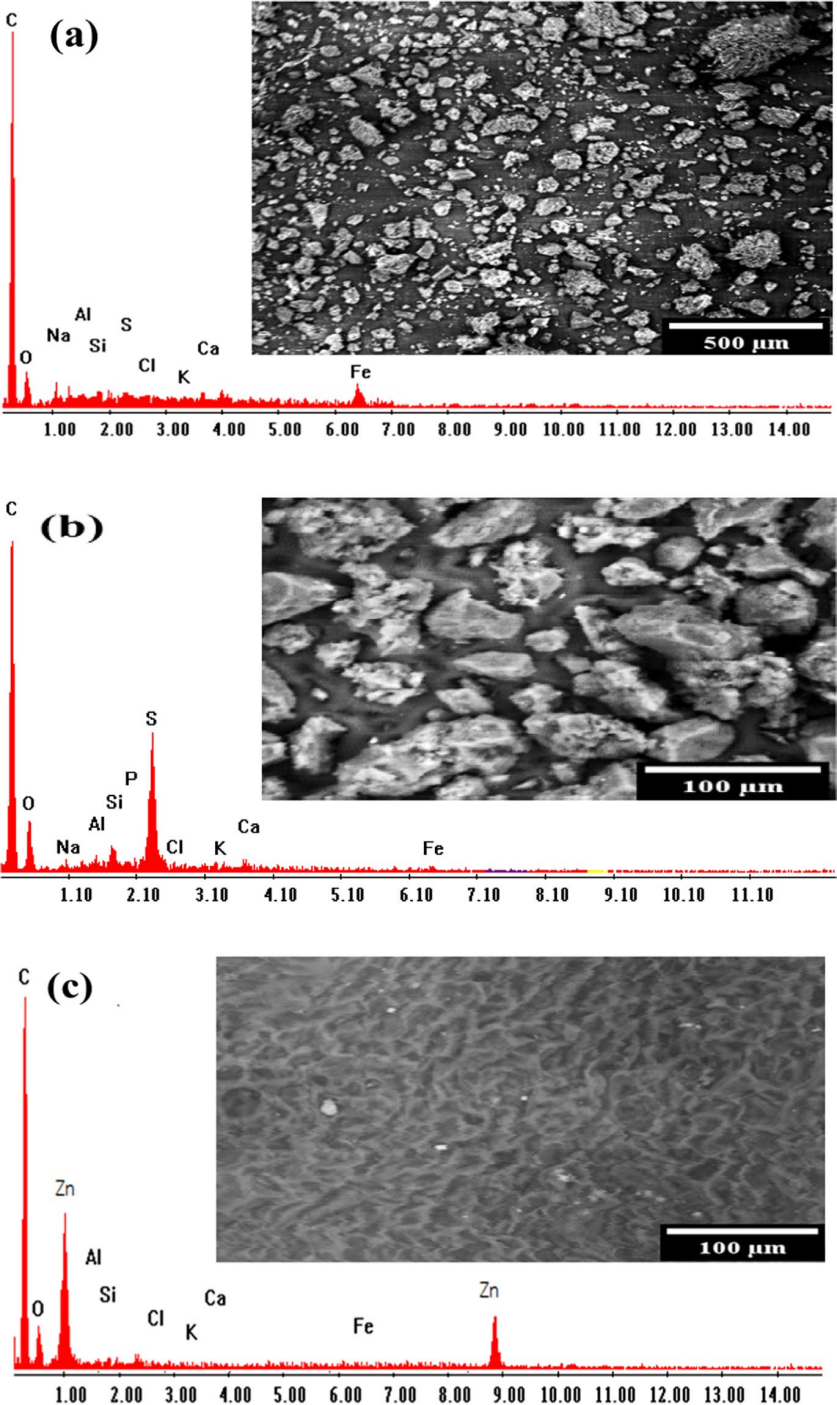
SEM and EDX images of the applied sorbents: **a** PBC, **b** PBC-SA, and **c** PBC-Zn

Scanning electron microscopy (SEM) and energy-dispersive X-ray analysis (EDX) techniques were used to investigate the surface morphology, elemental composition, and structural properties of the four different materials (Alhindawy et al., 2023). The anticipated results in Fig. 1 obvious that the three materials differ in their surface shape, with varying degrees of porosity, roughness, and pore structure. PBC displays a porous structure with irregular shapes and sizes (Fig. 1a). The surface appears rough and heterogeneous due to the presence of cracks and voids. SEM analysis for H_2_SO_4_-activated bio-char displays a more prominent pore network with an increased pore size distribution. The surface texture appears rougher than that of PBC, which indicates higher reactivity and sorption potential (Fig. 1b) (Kalyani et al., 2021). For PBC-Zn, it is clear that the activation process with ZnCl_2_ significantly changed the surface morphology. This material exhibits a highly porous structure with well-defined, interconnected pores. The surface appears to be relatively smoother and more structured when compared with PBC and PBC-SA, indicating the profound effect of ZnCl_2_ activation (Fig. 1c) (Liu et al., 2008).

**Fig. 2:**
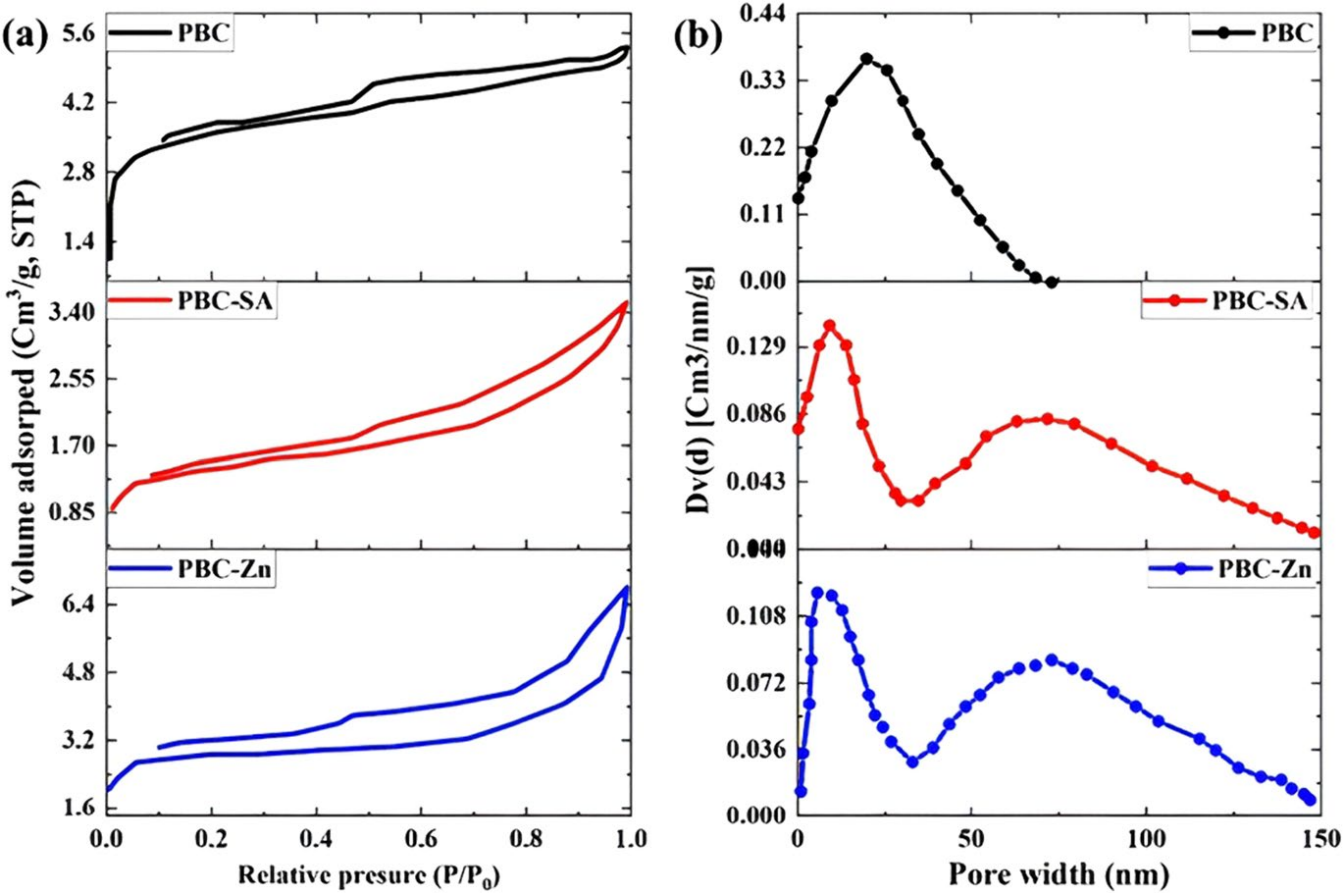
**a** N_2_ sorption-desorption and **b** the pore width of the PBC, PBC-SA, and PBC-Zn samples

Referring to the EDX analysis, the elemental compositions show differences due to the activation processes used in each material. Briefly, in the PBC sample (Fig. 1a), the primary element found is carbon (C), which is expected for a bio-char material. In addition, there are traces of oxygen (O) and other elements due to the raw materials and the carbonization process. The EDX analysis for PBC-SA sorbent (Fig. 1b) reveals a similar elemental composition to PBC, with slightly higher oxygen content and the emergence of sulfur (S). The elevated oxygen level may be a result of the activation process involving H_2_SO_4_, which likely introduced additional oxygen-based surface functional groups (Olivares-Marín et al., 2012). Lastly, in the case of PBC-Zn (Fig. 1c), carbon and oxygen are also detected, which is deemed to be due to the raw materials and the carbonization process. The distinctive feature is the presence of zinc (Zn) as a trace element, resulting from the activation process using ZnCl_2_.

By comparison, all three materials differ in their surface shape, with varying degrees of porosity, roughness, and pore structure. Moreover, the elemental compositions also show differences due to the activation processes used in each material. These differences indicate that activation agent selection plays an important role in modulating the surface properties and elemental composition of palm biomaterials.

#### BET

The N_2_ sorption–desorption isotherms provide valuable insights into the mesostructured nature of the three palm charcoal samples (Li et al., 2023). The overarching isotherm shape analysis (Fig. 2a) indicates all samples exhibit Type IV classification, with prominent hysteresis loops corresponding to type H3. These characteristic features signify the samples are mesoporous and contain slit-shaped pores, likely arising from stacked platelet-like construction. This trait appears intrinsic to the undifferentiated sample matrix, as the Type IV-H3 profile is conserved in both the raw bio-char and chemically activated variants. However, while the fundamental pore architecture is analogous, pore width characterization reveals notable activation-induced differences amongst the samples’ porosity (Fig. 2b).

The plain palm bio-char (PBC) possesses a narrowly distributed, predominantly microporous structure spanning just 0–70 nm. By stark contrast, the chemically activated PBC-SA and PBC-Zn samples display significantly broadened dual pore width distributions encompassing wider micro and mesopore ranges from 0–50 to 50–150 nm. Correspondingly, chemical activation generates appreciable mesopore development beyond the native micropore content of the source palm charcoal, forming a hierarchical bi-modal pore system. These additional mesopores augment diffusion and accession to the pore network, improving adsorptive performance(Sinha et al., 2019). Quantification of total pore volumes further substantiates porosity enhancement via chemical activation, with stepwise increases from unactivated PBC (5.4 cc/g) to sulfuric acid PBC-SA (6.8 cc/g) to zinc chloride PBC-Zn (7.1 cc/g). Additional pore size distribution analysis could pinpoint the specific locations of porosity amplification within the dual micro-mesopore structure induced upon activation (Li et al., 2020). The distinctions between the three materials regarding surface area, average pore width, and average pore size are presented in Table 1 (Khoshraftar et al., 2023).

**Table 1:** Surface characteristics for the PBC, PBC-SA, and PBC-Zn samples

Sample	Surface area (m^2^g^−1^)	Mean pore diameter (nm)	mean pore volume (cc/g)
PBC	802.086	34.93	5.4
PBC-SA	872.438	48.42403	6.8
PBC-Zn	880.273	50.4	7.1

#### Zeta potential

**Fig. 3:**
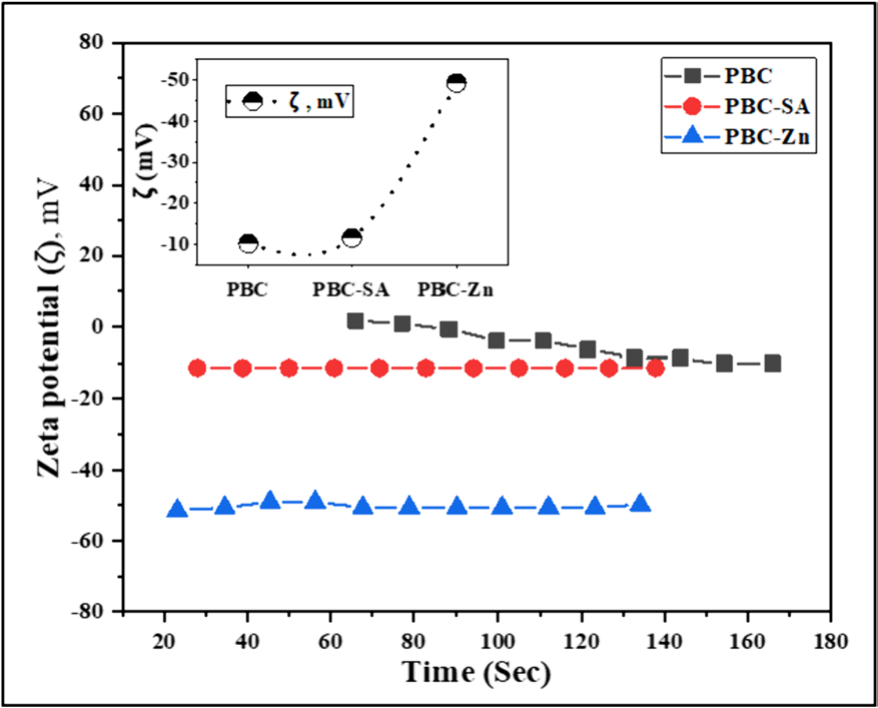
Zeta potential of the PBC, PBC-SA, and PBC-Zn samples

Zeta potential serves as a critical concept in surface science, influencing both the surface charge and stability of particles in liquids (Alhindawy et al., 2022a, 2022b; Dong et al., 2022). Zeta Potential analysis was performed for the three materials under study. Comparing the zeta potentials of the three studied materials shows significant differences (Fig. 3). PBC exhibits slightly negative values (− 10.11 mV), indicating moderate stability. PBC-SA is more negative (− 11.47 mV), likely due to the introduced negatively charged sulfate/sulfonic acid groups improving stability. PBC-Zn displays highly negative values (− 49.31 mV) probably due to zinc oxide species providing high negative charge and dispersion. Changes in zeta potential give insight into functional groups formed during activation and mechanisms influencing material stability(Alhindawy et al., 2022a, 2022b; Punnoose et al., 2014).

#### FTIR

**Fig. 4:**
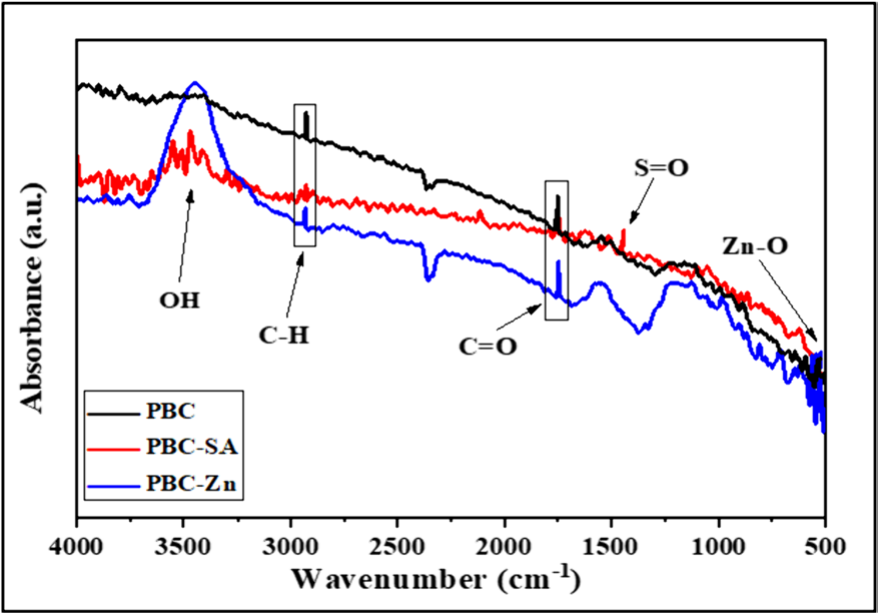
FTIR spectra of the PBC, PBC-SA, and PBC-Zn samples

FTIR spectroscopy was employed to analyze three sorbents derived from palm bio-char (Fig. 4). The obtained spectra revealed a broad band at 3200–3500 cm^−1^, attributable to ^−^OH from H_2_O or phenolic groups, indicating changes in the organic composition of the bio-char by the activation process (Eldoma et al., 2024; Geleto et al., 2022). The FTIR spectrum for PBC sorbent exhibited several peaks corresponding to various functional groups, such as C-H stretching vibrations at approximately 2927 cm^−1^, C = O stretching vibrations at around 1736 cm^−1^, and O–H stretching vibrations at approximately 3440 cm^−1^ (Bayuo et al. 2019b). Interestingly, PBC-SA induced changes in the FTIR spectrum, indicated by the appearance of a peak near 1248 cm^−1^ (Fankhauser et al., 2022), suggesting the presence of sulfate groups (S = O) and successful activation of PBC with H_2_SO_4_. These sulfate groups enhanced the bio-char’s surface polarity and increased its sorption capacity. The FTIR spectrum of the PBC-Zn exhibited a peak around 670 cm^−1^, indicative of Zn–O stretching vibrations (Sastry & Rao, 2015), implying the formation of zinc oxide (ZnO) on the surface of PBC, further enhancing its sorption properties. In PBC, the intensity of most organic functional bands significantly reduced after exposure to heat, hindering the formation of certain aromatic structures but increasing the surface area. Following the activation process in different media, the remaining aromatic structures were eliminated, as evidenced by the emergence of a new peak at 2362 cm^−1^ in the two activated materials, thereby further increasing the surface area compared to unactivated PBC.

### Equilibrium studies

#### Effect of pH

**Fig. 5:**
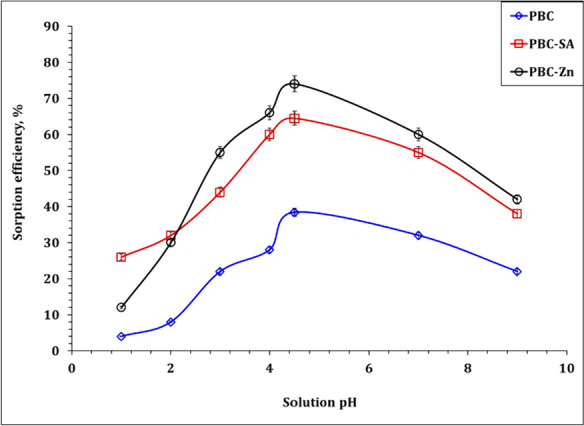
Effect of pH on the sorption efficiency of PBC-Zn, PBC-SA, and PBC

Solution pH significantly impacts the chemistry of the metal ions and the sorbent surface charge as well, therefore, it is important for the sorption process. In this regard, a set of experiments was conducted using different pH values ranging from 1.0 to 9.0, while other conditions were kept constant at room temperature, sorbent dose of 2.5 g/L, U(VI) initial concentration of 50 ppm, and shaking time of 120 min. Figure 5 declares that the sorption capacity of PBC, PBC-Zn, and PBC-SA for U(VI) species reaches its peak at pH 4.5. At low pH, the high concentration of H^+^ ions in the acidic solution competes with U(VI) ions for the sorbent surface-free sites (Hu et al., 2023). As the pH gradually increases from 1.0 to 4.5, the concentration of H^+^ ions in the solution decreases, which gives more chance for the interaction between U(VI) ions and sorbent active sites. However, when the pH exceeds 4.5, the sorption capacity is dramatically decreased which is deemed to be attributed to the presence of the uranium insoluble species (UO_2_(OH)_2_⋅H_2_O)(Wang et al., 2009). This finding aligns with similar observations made by many previous studies(Baby et al. 2019, 2023).

#### Effect of contact time

**Fig. 6:**
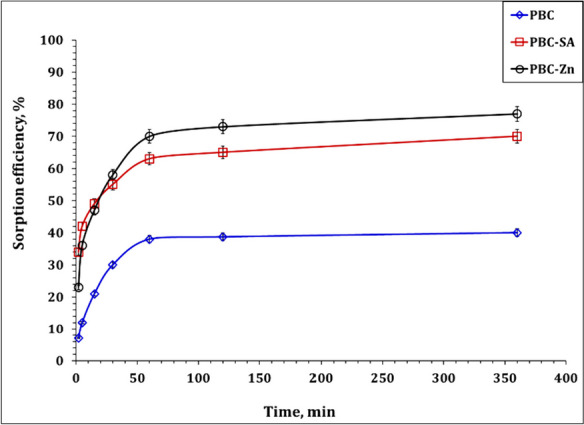
Effect of contact time on the sorption efficiency of PBC-Zn, PBC-SA, and PBC

The crucial factor in achieving equilibrium in the sorption process is the contact time. In this context, to evaluate the impact of contact time on the U(VI) sorption using PBC, PBC-SA, and PBC-Zn sorbents, a group of tests were performed at contact time, ranging from 2 to 360 min (Fig. 6), while maintaining all other parameters constant at pH 4.5, sorbent dose of 2.5 g/L, U(VI) initial concentration of 50 ppm and room temperature. It is evident from the results that there is an initial rapid increase in sorption efficiency for PBC (38.0%), PBC-SA (63.0%), and PBC-Zn (70.0%) ions within the first 60 min (equilibrium state), indicating a swift sorption rate during this period. This phenomenon can be attributed to the initial abundance of vacant active sites available for uranium ions at the outset of the sorption process (Bayuo, 2021b). However, beyond the 60 min, the graph stabilizes, and there is no significant further enhancement in the sorption percentage. This stabilization occurs because the active sites on the prepared adsorbent became saturated with adsorbed ions, resulting in no notable increase in sorption efficiency. This observation aligns with a study conducted by Çakır et al. (2014)(Cakir et al., 2014) on U(VI) ions sorption from aqueous solution on zirconium-antimony oxide/polyacrylonitrile (Zr–Sb oxide/PAN).

#### Effect of the sorbent dosage

**Fig. 7:**
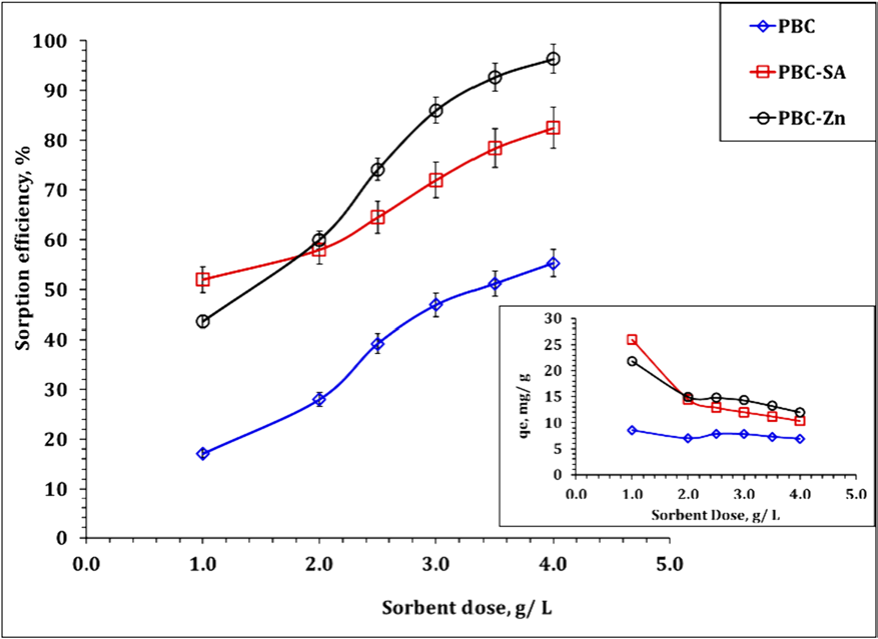
Effect of adsorbent dosage on the sorption efficiency of PBC-Zn, PBC-SA, and PBC

It is crucial to investigate how changing the adsorbent dose from 1.0 to 4.0 g/L and maintaining constant conditions of initial uranium concentration of 50 ppm, pH 4.5, sorption time of 120 min, and room temperature will affect the sorption capacity and removal efficiency of PBC, PBC-Zn, and PBC-SA for uranium(VI). Figure 7 displayed the increment of the U(VI) sorption percent as the adsorbent dosage rose from 1.0 to 4.0 g/L. The surge in active sites and the area in contact with uranium(VI) can be blamed for this increase(Bayuo et al., 2019). In contrast, the sorption capacity declined with the increment of the sorbent dose which, is probably because less uranium(VI) was absorbed by each unit mass of material as the adsorbent dosage rose (Bayuo et al. 2022c). This finding is consistent with several research investigations (Hussein et al., 2019; Morsy et al. 2019b), All of these studies focused on the sorption of U(VI) from aqueous solution.

#### Effect of the initial concentration

**Fig. 8:**
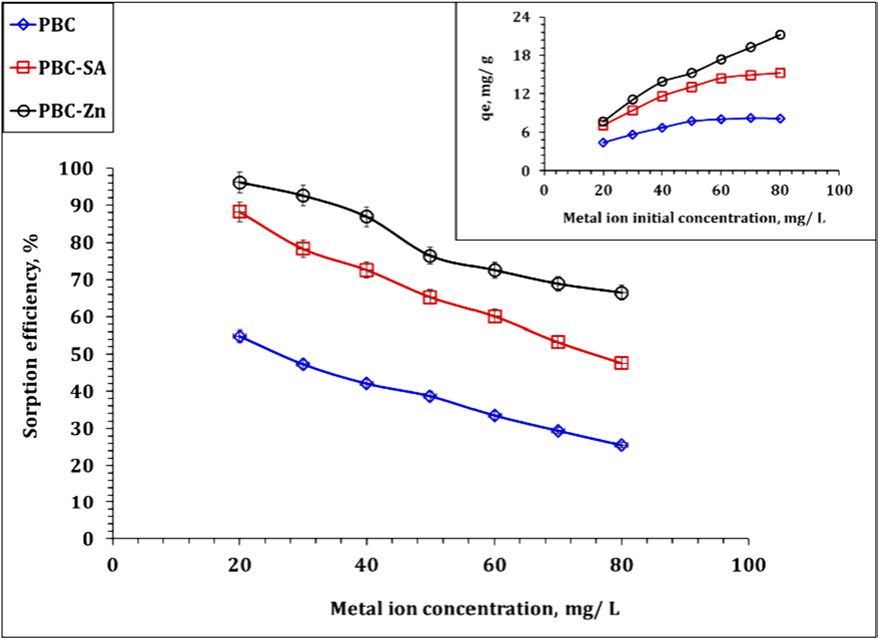
Effect of initial concentration on the sorption efficiency of PBC-Zn, PBC-SA, and PBC

By submitting the bio-char adsorbents PBC, PBC-Zn, and PBC-SA to a constant sorbent dose of 2.5 g/L, the effect of the initial U(VI) content on the sorption rate was investigated. This experiment had a contact time of 120 min, a starting pH of 4.5, and was carried out at room temperature. The initial U(VI) concentrations in the experiment varied from 20 to 80 ppm. All PBC, PBC-Zn, and PBC-SA sorbents display the same sorption performance, as demonstrated by the results in Fig. 8, whereas as the starting concentration increases, the sorption efficiency drops but the sorption capacity increases. This is because the ions are effectively adsorbed onto the adsorbent at low concentrations, leading to a rapid removal rate. The available active sites for adsorbing the ions, however, become saturated as the ion concentration rises, resulting in a decline in the removal rate. In addition, an increase in uranium ions causes an increase in the amount of adsorbed material due to an increase in the sorption capacity per unit area (Hussein et al., 2019; Morsy et al. 2019b). The reported literature makes up this performance(El-Maadawy, 2019; Youssef et al., 2022).

### Sorption modeling

#### Sorption kinetics

**Fig. 9:**
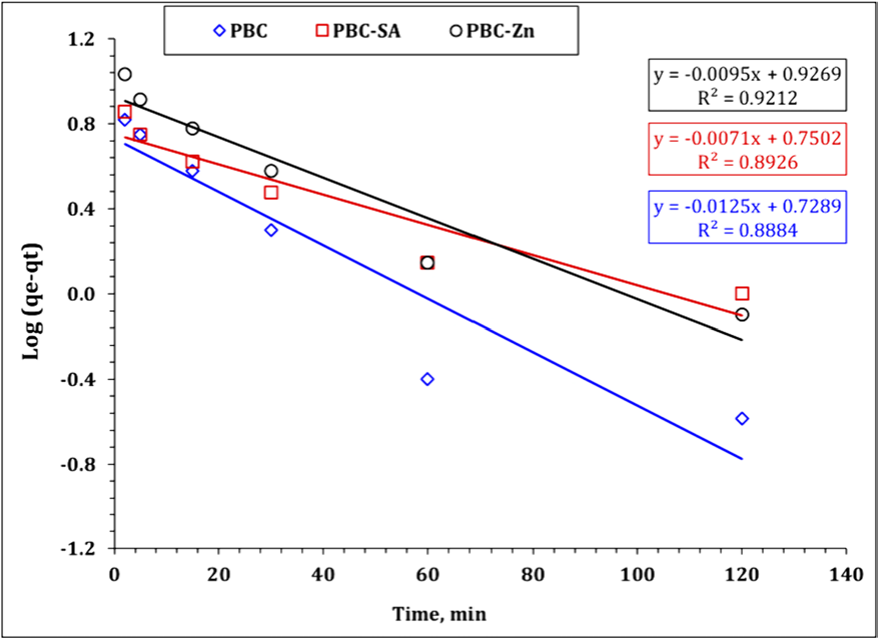
Pseudo first-order plot for uranium sorption using PBC-Zn, PBC-SA, and PBC

**Fig. 10:**
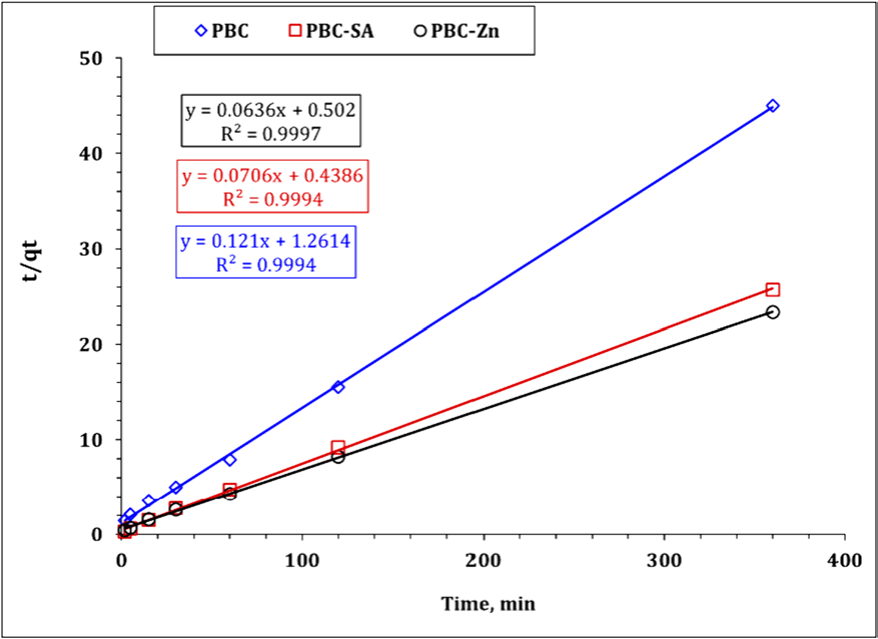
Pseudo second-order plot for uranium sorption using PBC-Zn, PBC-SA, and PBC

**Fig. 11:**
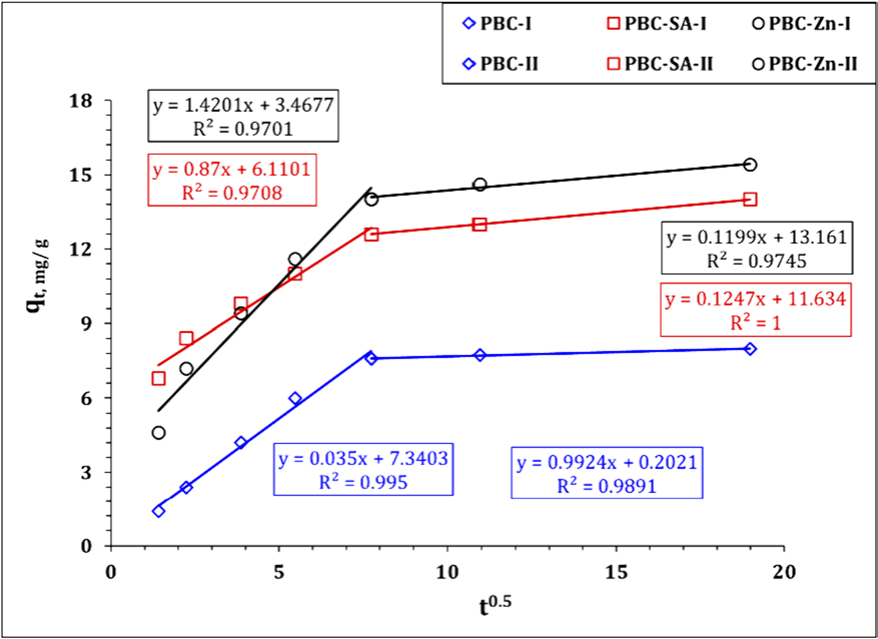
Intraparticle diffusion plot for U(VI) sorption using PBC-Zn, PBC-SA, and PBC

The kinetic investigation’s findings reveal the sorption process’s physical or chemical nature, the sorption mechanism, and the rate-controlling step (Kang & Kim, 2019; Wu et al. 2009). Pseudo-first order (PFO), Pseudo-second order (PSO), and intraparticle diffusion (IPD) kinetic models (Bayuo et al., 2020, 2023b; Kang & Kim, 2019) were used to figure out the kinetics of uranium sorption from aqueous solution onto bio-char adsorbents PBC, PBC-Zn, and PBC-SA. Table S1 declares the linear forms of the applied models (Bayuo et al., 2020, 2023b; Kang & Kim, 2019). The values of model terms can be achieved from the linear plots of ln (*q*_*e*_–*q*_*t*_) versus *t* ( PFO plot), *t*/*q*_*t*_ versus *t* (PSO plot), and the slope of the *q*_*t*_ vs *t*½ (IPD plot) (El-Sabbagh et al., 2023). Figures 9, 10, and 11 represent the kinetic plot of the PFO, PSO, and RID kinetic models respectively. The kinetic parameters were evaluated from these kinetic plots and attained in Table 2.

**Table 2:** Kinetics parameters for uranium sorption using PBC-Zn, PBC-SA, and PBC

		**PBC**	**PBC-SA**	**PBC-Zn**
**Pseudo first-order**	*k*_1_ (min^−1^)	0.029	0.016	0.022
	*qe*_cal_ (mg/g)	5.4	5.6	8.5
	*qe*_exp_ (mg/g)	8.0	14.0	15.4
	*R*^2^	0.89	0.89	0.92
**Pseudo second-order**	*k*_2_ (min^−1^)	0.012	0.011	0.008
	*qe*_cal_ (mg/g)	8.3	14.2	15.7
	*qe*_exp_ (mg/g)	8.0	14.0	15.4
	*h* (mol g^−1^ h^−1^)	0.79	2.28	1.99
	*t*_1/2_ (h)	10.4	6.2	7.9
	*R*^2^	0.99	0.99	0.99
**Weber and Morris model**	**Stage I**			
	*k*_*i*_ (mg/g min^1/2^)	0.70	0.64	1.03
	*C*	1.2	6.9	4.8
	*R*^2^	0.99	0.97	0.97
	**Stage II**			
	*k*_*i*_ (mg/g min^1/2^)	0.03	0.12	0.10
	*C*	7.4	11.6	13.5
	*R*^2^	0.99	0.99	0.97

The attained data declares that the Pseudo second order kinetic equation exhibits the highest correlation coefficient for all applied bio-char materials (*R*^2^ = 0.99). Furthermore, the evaluated uptake affinity *qe*_cal_ for carbonaceous materials PBC (8.3 mg/g), PBC-SA (14.2 mg/g) and PBC-Zn (15.7 mg/g) are close to the experimental sorption capacities qe_exp_ ( PBC: 8.0 mg g^−1^; PBC-SA: 14.0 mg g^−1^; PBC-Zn: 15.4 mg g^−1^) which means that the uranium uptake process using the prepared bio-char materials PBC, PBC-SA, and PBC-Zn is described well using Pseudo second order kinetic models. This declares the chemisorption nature of the sorption process and the uranium uptake involves the sharing of electrons between uranium species and the active sites of the applied carbons (Abdel-Magied, 2017; Morshedy et al., 2021; Mohamed H. Taha et al., 2019). This kinetic performance (chemisorption nature) for U(VI) removal is also reported in many studies (Abdel-Magied, 2017; Morshedy et al., 2021; Morsy et al. 2019c). The sorption capacity could be ranked as PBC-Zn > PBC-SA > PBC which reflects that the increase in surface area enhances the sorption characteristics of the yield carbons. The enhancements can be attributed to the creation of micropores and mesopores, the removal of volatile matter, and the restructuring of the carbon matrix (Morshedy et al., 2021).

The intraparticle diffusion model (IPD) proposed by Weber and Morris has been widely applied for exploring the interaction mechanism between U(VI) and the applied sorbents. So far the U(VI) sorption reaction is controlled with a solo mechanism in case the plot of *qt* as a function of time^0.5^ (IPD plot) yields a linear relationship passing through the origin (one line segment) otherwise, the sorption reaction is controlled with multiple mechanisms if the plot yields numerous line segments. The IPD plot (Fig. 11) reveals two distinct segments, indicating that various techniques were employed to control the sorption of uranium ions using PBC, PBC-SA, and PBC-Zn. The first mechanism may involve chemical reactions, such as surface complexation or chelation reactions, which are pertinent to the sorption process up to the equilibrium time of 60 min. This initial stage is marked by a rapid sorption reaction, likely due to the availability of surface active sites on the produced bio-char materials (Abdel-Magied, 2017; Morshedy et al., 2021; Morsy et al. 2019c). The second mechanism, the intraparticle diffusion mechanism, which describes the sorption process following equilibrium, may entail a physical response. Due to the occupation of the majority of surface active sites, a slow reaction rate is seen at this stage. As a result, the sorption process takes place inside the surface pores (El-Sabbagh et al., 2023). In other research, the same various response mechanisms were mentioned (Abdel-Magied, 2017; Morshedy et al., 2021; M. H. Taha et al., 2018).

#### Sorption isotherm

Sorption isotherms show the relationship between the concentration of a solute in the solid phase and the aqueous phase at a given temperature. In the present work, three isotherm models (i.e., Freundlich, Langmuir, and Temkin models) were applied to investigate the isotherm of U(VI) sorption using the generated bio-char materials (PBC, PBC-SA, and PBC-Zn). Freundlich model gives insight into the heterogeneous, multilayer sorption; the Langmuir model assumes the monolayer sorption; the Temkin model describes the sorption heat (Bayuo et al., 2020, 2023b; Kim & Kim, 2019). Table S1 declares the linear forms of the applied models (Bayuo et al., 2020, 2023b; Kim & Kim, 2019).

**Fig. 12:**
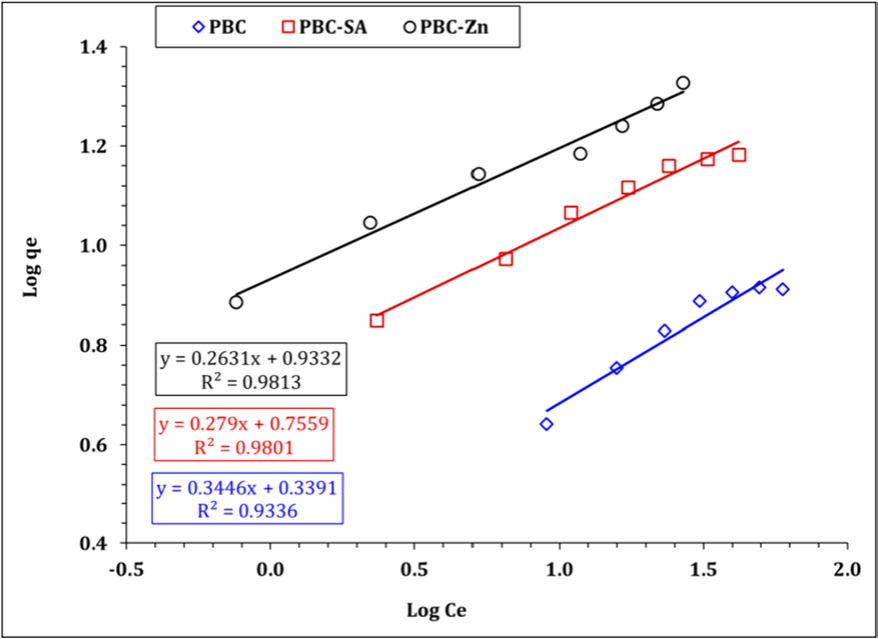
Freundlich plot for uranium sorption using PBC, PBC-Zn, and PBC-SA

**Fig. 13:**
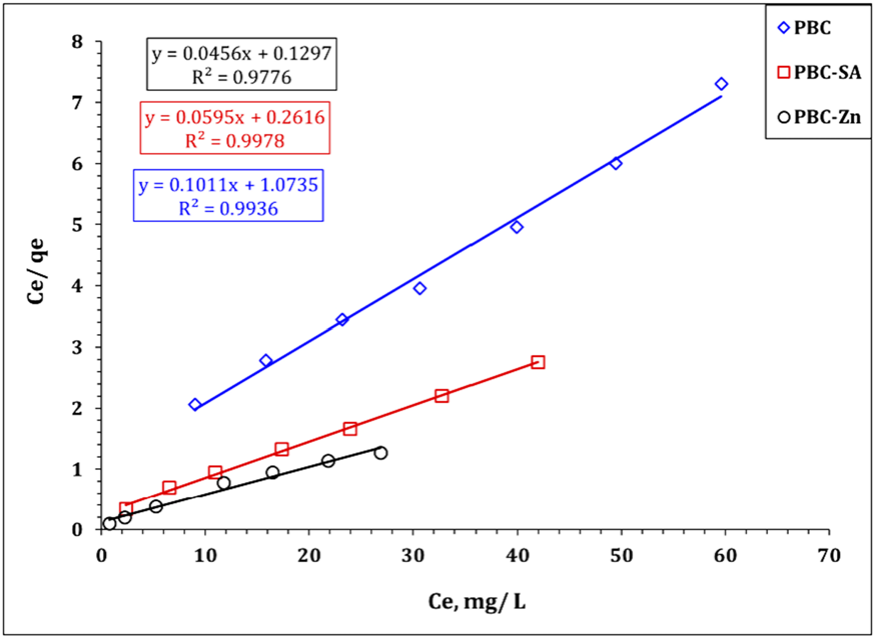
Langmuir plot for uranium sorption using PBC, PBC-Zn, and PBC-SA

Freundlich isotherm variables could be explored by the illustration of log *q*_*e*_ against the log *C*_*e*_ (Fig. 12), while the relation between *C*_*e*_/*q*_*e*_ versus *C*_*e*_ (Fig. 13) could be used for the evaluation of Langmuir isotherm variables. Furthermore, the Langmuir parameters can be used to predict the affinity between the sorbate and adsorbent utilizing the dimensionless separation factor *R*_*L*_ (El-Sabbagh et al., 2023):

4 $${R}_{L}=\frac{1}{1+ {K}_{L}{C}_{0}}$$

where *C*_0_ is the initial concentration of uranium ions. Values of 0 < *R*_*L*_ < 1 expose the nature of sorption as favorable.

The estimated isotherm variables are displayed in Table 3. The anticipated isotherm results obvious that the uranium species illumination obeyed to Langmuir isotherm model since it possesses the highest correlation coefficient (*R*^2^ = 0.99) for all applied sorbents which reflects a monolayer and homogeneous sorption process (Bayuo et al., 2020, 2023b; Kim & Kim, 2019). Furthermore, as illustrated in Figure S2, all *R*_*L*_ (dimensionless separation factor) values for the sorption of uranium ions onto the prepared bio-char materials (PBC, PBC-SA, and PBC-Zn) fall within the range of zero to one, even when considering a wide initial uranium concentration range of 20 to 80 mg L^−1^. This finding strongly implies that the sorption of U(VI) on these bio-char materials occurs under very favorable conditions. It further confirms that, regardless of the initial uranium concentration, the sorption of uranium onto PBC, PBC-SA, and PBC-Zn is not only effective but also preferable (El-Sabbagh et al., 2023).

**Table 3:** The evaluated variables of Langmuir, Freundlich, and Temkin isotherm models

		**PBC**	**PBC-SA**	**PBC-Zn**
**Freundlich isotherm model**	*n*	2.9	3.6	3.8
	*K*_*f*_ (mg/g)	2.18	5.7	8.6
	R^2^	0.93	0.98	0.98
**Langmuir isotherm model**	*q*_*m*_ (mg/g)	9.89	16.8	21.9
	*b* (L/mg)	0.094	0.23	0.35
	R^2^	0.99	0.99	0.98
**Temkin isotherm**	*b* (J/mol)	1144.4	819.3	709.8
	B	2.2	3.0	3.5
	*K*_*T*_ (L/g)	0.9	4.1	10.6
	*R*^2^	0.95	0.99	0.96

**Fig. 14:**
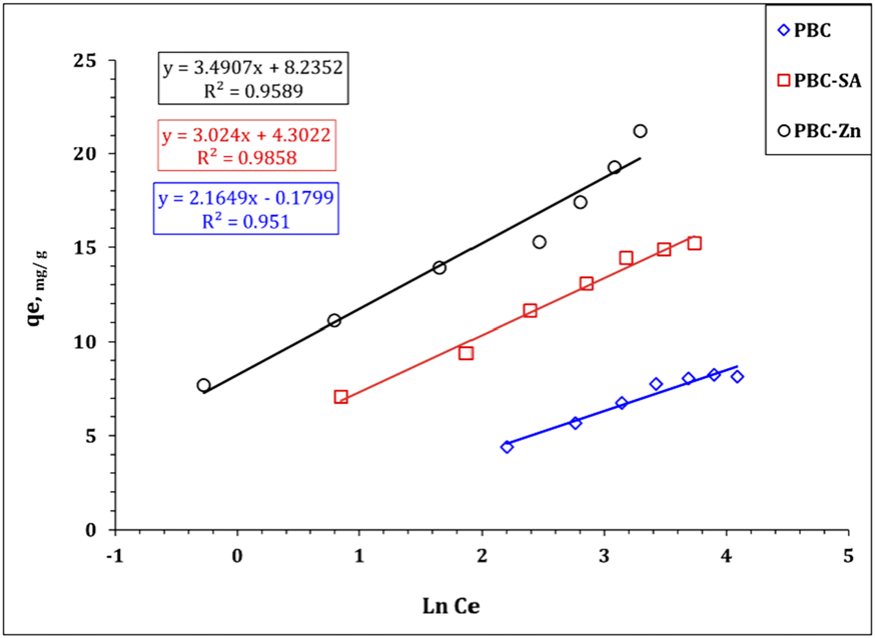
Temkin model plot for uranium sorption using PBC, PBC-Zn, and PBC-SA

It worth noted that the Freundlich isotherm model (Table 3) exhibits a proper coordination coefficient (*R*^2^ =  > 0.95) which deduced that the multi-layer sorption process could also contribute to U(VI) uptake using blank and activated sorbents particularly at low initial concentration (Bayuo et al., 2020, 2023b; Kim & Kim, 2019). This reflects that U(VI) uptake process is mainly a monolayer process with a contribution to the multi-layer sorption process to a limited extent. The same isotherm profile for U(VI) removal was reported(Abdel-Magied, 2017; Morshedy et al., 2021; Taha et al., 2018). In addition, Table 3 declares that Temkin isotherm model possesses a good coordination coefficient (0.98), confirming a uniform and ideal adsorbent surface which is consistent with the findings of Langmuir isotherm model. The nature of the sorption process could be explored from the sign and magnitude of the b_T_ value whereas the positive *b*_*T*_ values (1144.4, 819.3, and 709.8 J mol^−1^ for PBC, PBC-SA, and PBC-Zn, respectively) elucidated an exothermic and physisorption (less than 8 kJ mol^−1^) nature for the U(VI) sorption process. So far, the high *b*_*T*_ value means a high negative impact with the boost of reaction temperature, which indicates that the negative impact of reaction temperature increment on U(VI) sorption efficiency could be ranked as PBC > PBC-SA > PBC-Zn. This is consistent with the experimental data in Fig. 14. Table 3 displayed that the maximum binding energy for PBC, PBC-SA, and PBC-Zn are 0.9, 4.1, and 10.6 L g^−1^ respectively which illuminated more affinity for the sorbent towards U(VI) ions could be ranked as PBC-Zn > PBC-SA > PBC which is consistent with the finding of the experimental results (Fig. 14).

The maximum U(VI) sorption capacities of the PBC-PA, PBC-Zn, and PBC-SA sorbents were compared with those of other sorbents reported in the literature, as presented in Table 4. The results indicate that the sorption capacities of the employed sorbents align closely with those of the sorbents cited in the literature which reflects that the prepared three sorbents have all demonstrated effective performance, making them suitable choices for established hydrometallurgy applications.

**Table 4:** U(VI) sorption results reported in the literature

Sorbent	*q*_*m*_, mg g^−1^	Ref
Blank bio-char(Bc)	2.30	(Morshedy et al., 2021)
Hydrochloric acid-activated carbon(NAC)	2.30	
Metalized bio-char (MBC)	3.10	(Ali et al., 2020)
DEDTC-PVC/DEHPA/Fe@C nanoparticles	3.36	(Bromberg et al., 2017)
Eucalyptus wood bio-char	27.2	(Mishra et al., 2017)
Fungus Pleurotus ostreatus	19.4	(Zhao et al., 2016)
Pistacia vera L. shell-activated carbon	8.6	(Donat & Erden, 2017)
Hazelnut shell-activated carbon	16.3	(Zhu et al., 2016)
Agricultural carbon fiber CF-RH	21.05	(Abou-Hadid et al., 2022)
Agricultural carbon fiber CF-SCB	29.15	
PBC	9.89	**Pw**
PBC-SA	16.81	**Pw**
PBC-Zn	21.93	**Pw**

*Pw present work

**Fig. 15:**
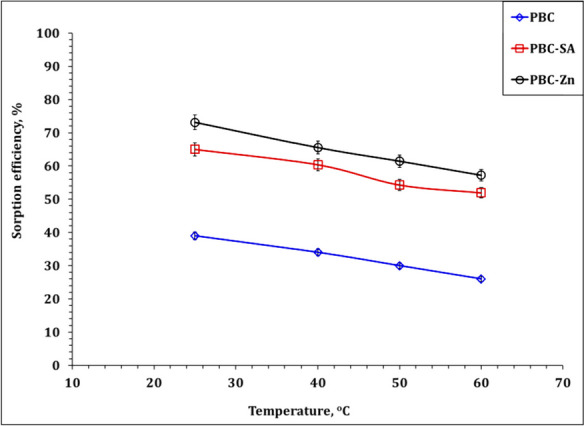
Effect of temperature on the sorption efficiency of PBC-Zn, PBC-SA, and PBC

Temperature plays a significant role in influencing the rate of ion diffusion and the sorption capacity of the adsorbent in reaching sorption equilibrium. As demonstrated in Fig. 15, when maintaining a pH of 4.5, a sorbent dose of 2.5 g/L, a contact time of 120 min, and an initial uranium concentration of 50 ppm, the sorption efficiency of the adsorbent experiences a slight decrease with rising temperature within the range of 25–60 °C. This suggests that, in this temperature range, lowering the temperature enhances the sorption of U(VI) ions by all the bio-char materials, namely PBC, PBC-SA, and PBC-Zn.

The thermodynamic equations displayed in Table S1 (El-Sabbagh et al., 2023) were applied to assess the thermodynamic variables, i.e., standard free energy change (∆G^o^, kJ/mol), standard enthalpy change (∆H^o^, kJ/mol), and standard entropy change (∆S^o^, J/(mol. K)). An illustration of log Kc *VIS* 1/T is displayed in Figure S3, while the values of the thermodynamic parameters are exhibited in Table 5.

**Table 5:** Thermodynamic parameters for uranyl ions by PBC, PBC-Zn, and PBC-SA

Adsorbent	Δ*G* (kJ/mol)	Δ*H* (kJ/mol)	Δ*S* (J/mol K)
PBC	− 13.73 ± 0.04	− 13.60 ± 0.02	0.44 ± 0.01
PBC-SA	− 16.38 ± 0.06	− 13.34 ± 0.04	10.17 ± 0.02
PBC-Zn	− 17.33 ± 0.07	− 16.74 ± 0.05	1.97 ± 0.03

The data presented in Table 5 suggests that the sorption process is exothermic, as evident from the negative values of ΔH^o^, which are − 13.60 kJ mol^−1^, − 13.34 kJ mol^−1^, and − 16.74 kJ mol^−1^ for PBC, PBC-SA, and PBC-Zn, respectively. The negative values of Gibbs free energy (ΔG^o^) indicate that the sorption process is both spontaneous and feasible(Khawassek et al., 2018). The positive values of ΔS^o^ for the uptake process recommend an increase in randomness at the solid–liquid interface (Orabi et al., 2021). Similar thermodynamic behavior (i.e., exothermic, spontaneous, and feasible sorption process) was observed in the sorption of uranium ions from aqueous solution in previous studies (Khawassek et al., 2018; Orabi et al., 2021).

#### Desorption and reusability investigation

The elution of uranium from the loaded PBC-Zn (which gives the heist sorption capacity) was studied using various solutions, including H_2_SO_4_, HNO_3_, HCl, CH_3_COONa, and Na_2_CO_3_. Desorption experiments were conducted by shaking 3.5 g/L of the loaded PBC-Zn sample with the eluent solution (1 M). The elution efficiency was systematically calculated and is presented in Table S2. The data obtained indicates that the CH_3_COONa solution exhibits the highest elution efficiency (92.0%). The reusability of the prepared PBC-Zn sorbent was investigated by performing five sorption/desorption sequence cycles. Table S3 obvious that the sorption and desorption percent slightly changed from 92.6 to 89.6% for the sorption process, and from 91.4 to 88.4% for the desorption process over the five cycles, which reflects the feasibility of sorbent recycling.

#### Uranium removal from the liquid raffinate solution (Case study)

From an environmental perspective, the method of removing uranium from wastewater is crucial. As a result, an application experiment was carried out to recover uranium ions from a liquid waste solution produced by the Nuclear Materials Authority, Egypt, using the PBC-Zn sorbent. The main chemical composition of the real sample was 1.9 ppm Ca(II), 0.60 ppm Fe(III), 50 ppm of uranium(VI) as measured spectrophotometrically using thiocyanate method at *λ*_max_ = 495 nm for iron ions, Glyoxal bis(2-hydroxyanil) method at *λ*_max_ = 516 nm for calcium ions, and using Arsenazo III method at *λ*_max_ = 665 nm for uranium ions (Marczenko & Balcerzak, 2000). The testing conditions included a pH of 3.1, 3.5 g/L of PBC-Zn addition, 120 min of mixing duration, and room temperature. According to the findings, the uranium content of the raffinate sample was successfully eliminated to a degree of about 93%. With an emphasis on its potential for environmental applications, this result implies that using PBC-Zn sorbent is a promising and practical choice for the process of remediating liquid waste.

## Conclusion

Palm kernel shells, a type of solid waste residue, underwent pyrolysis to produce bio-char species (PBC), which were then utilized to create two activated carbon structures using sulfuric acid (PBC-SA) and zinc oxide (PBC-Zn). The resulting bio-char products underwent a comprehensive analysis through Scanning Electron Microscopy (SEM), Energy X-ray Dispersion (EXD), Brunauer–Emmett–Teller (BET), Fourier Transform Infrared Spectroscopy (FTIR), and Zeta potential tests. The sorption isotherms revealed a Langmuir model pattern, with sorption capacities of 9.89, 16.8, and 21.9 mg/g for PBC, PBC-SA, and PBC-Zn, respectively. These sorption capacities were observed under specific operational parameters: a solution pH of 4.5, a contact time of 120 min between the adsorbent and U(VI) solution, an initial U(VI) concentration of 50 ppm, the adsorbent dosage of 2.5 g/L, and room temperature. Additionally, the introduced sorption data fittingly adhered to the pseudo-second-order kinetic model. Thermodynamic analysis indicated that uranium removal by the three structures is a viable, spontaneous, and exothermic process. Moreover, approximately 92% of uranium-loaded PBC-Zn sorbent was successfully eluted using a 1.0 M CH3COONa sodium ethanoate solution, demonstrating proper stability over five consecutive sorption/desorption cycles.

### Supplementary Information

Below is the link to the electronic supplementary material.

Supplementary file1 (DOCX 162 KB)

## Data Availability

The datasets used and/or analyzed during the current study are available from the corresponding author on reasonable request.
