# Supplementary material for: Uranium capture from aqueous solution using palm-waste based activated carbon: sorption kinetics and equilibrium
Source: Environ Monit Assess. 2024 Apr 4;196(5):428. doi: 10.1007/s10661-024-12560-y (PMC10995074; doi:10.1007/s10661-024-12560-y)
Supplement: Supplementary file 1 — Supplementary file1 (DOCX 162 KB) [file 10661_2024_12560_MOESM1_ESM.docx]

**Supplementary Information**


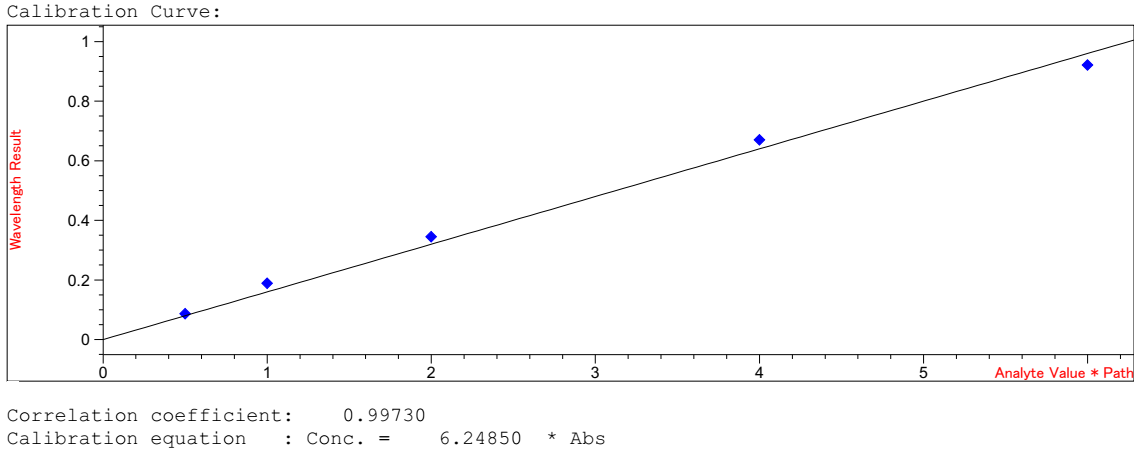


**Figure S1:** *Calibration curve for U(VI) determination using spectrophotometer at 654 nm*


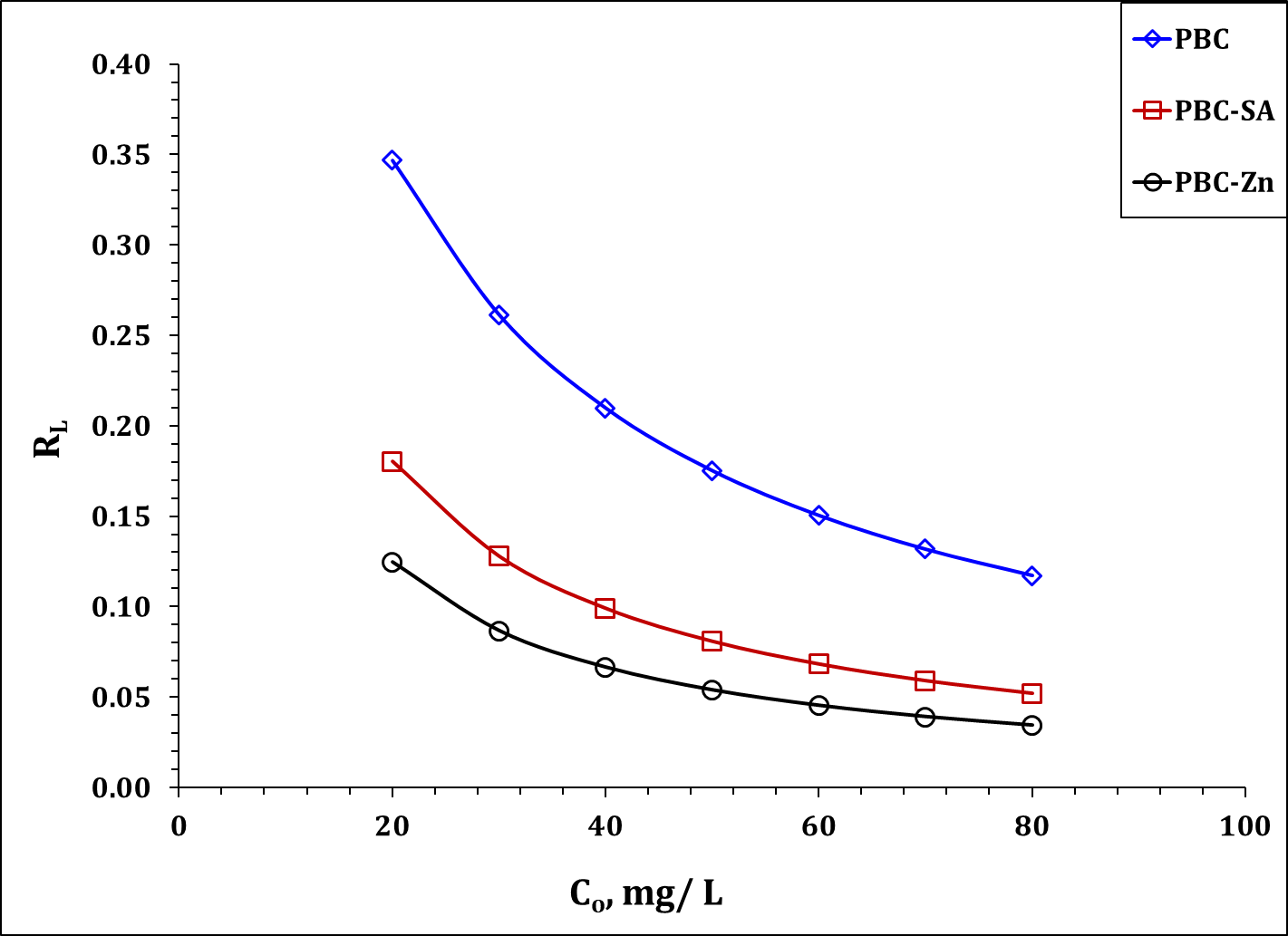


**Figure S2:** The R_L_ values for uranium sorption using PBC, PBC-Zn, and PBC-SA


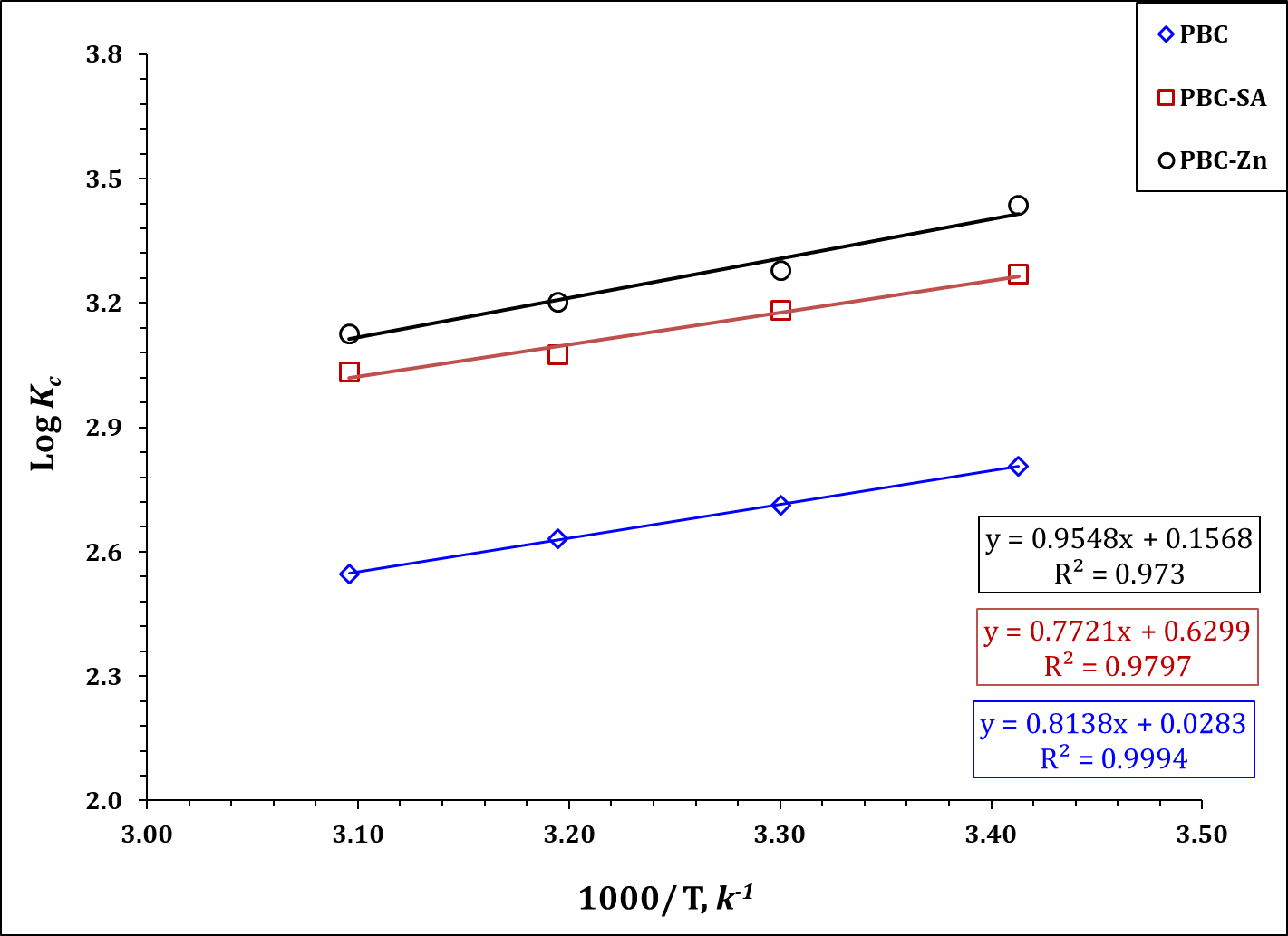


**Figure S3**: Van’t Hoff equation plot for uranium sorption using PBC, PBC-Zn and PBC-SA

**Table S1**: Kinetic, isotherm, and thermodynamic equations for the sorption of U(VI) ions using PBC-Zn, PBC-SA and PBC sorbents(Kang and Kim 2019; Bayuo et al. 2020, 2023b; El-Sabbagh et al. 2023; Kim and Kim 2019).

| Kinetics | Equations | |
| --- | --- | --- |
| Pseudo-first-order | $\mathrm{Log}\left( q_{e}-q_{t} \right)=Log q_{e} - \frac{K_{1}}{2.303} t$ | |
| Pseudo-second-order | $\left( \frac{t}{q_{t}} \right)=\frac{1}{K_{2}q_{e}^{2}}+ \frac{1}{q_{e}} t$ | |
| Intra-particle diffusion model (IPD) | $q_{t}= K_{id}t^{0.5} + C_{i}$ | |
| Isotherms | **Equations** | |
| Langmuir model | $\frac{C_{e}}{q_{e}}=\left( 1 \vert K_{L}q_{m} \right)+\left[ \frac{C_{e}}{q_{m}} \right]$ | |
| Freundlich model | | $\ln q_{e}=\ln K_{F}+\left[ \frac{1}{n} \right]\ln C_{e}$ |
| Temkin model | | $\boldsymbol{q}_{\boldsymbol{e}}\mathbf{=}\frac{\boldsymbol{RT}}{\boldsymbol{b}_{\boldsymbol{T}}}\mathbf{ln} \boldsymbol{K}_{\boldsymbol{T}}\boldsymbol{C}_{\boldsymbol{e}}$ |
| Thermodynamics | | **Equations** |
| ${\log\boldsymbol{K}}_{\boldsymbol{C}}\mathbf{=-}\frac{\boldsymbol{\Delta H}^{\boldsymbol{o}}}{\boldsymbol{2.303 R}}\mathbf{X}\frac{\boldsymbol{1}}{\boldsymbol{T}}\mathbf{+A}$ | | |
| $\mathbf{-}\boldsymbol{\Delta G}^{\boldsymbol{o}}\mathbf{=2.303 RT}\log\boldsymbol{K}_{\boldsymbol{C}}$ | | |
| $\boldsymbol{\Delta G}^{\boldsymbol{o}}\mathbf{=}\boldsymbol{\Delta H}^{\boldsymbol{o}}\mathbf{- T}\boldsymbol{\Delta S}^{\boldsymbol{o}}$ | | |

qe (mg g^-1^) is the equilibrium concentration of U(VI) ions , and qt (mg g^-1^) is the adsorbed amount of U(VI) ions after time t (min), k_1_ (min^-1^) and k_2_ (min^-1^) are the rate constants for the pseudo first and second order, respectively. K_id_ (mg/g. min^0.5^) is a rate constant, and C is the thickness of the boundary layer. Ce (ppm) is equilibrium concentration of U(VI) ions, q_max_ (mg g^-1^) is the theoretical adsorption capacity, K_L_ is Langmuir constant, K_F_ and n are Freundlich constants. *b_T_* is Temkin constant that refers to the sorption heat, and *K_T_* (L min^-1^) is the equilibrium binding constant. $K_{C}$ is a non-dimensional equilibrium constant and it equals K_d_ X 1000 X ρ **[5]**; $\boldsymbol{T}$ is the temperature (K), 𝑹 is the universal gas constant (8.314 J mol ^-1^. K^-1^), ρ is solution denisty g/ L, and $\boldsymbol{A}$ is a constant.

Bayuo J, Abukari MA, Pelig-Ba KB (2020b) Optimization using central composite design (CCD) of response surface methodology (RSM) for biosorption of hexavalent chromium from aqueous media. Appl Water Sci 10:1–12.

Bayuo J, Rwiza MJ, Mtei KM (2023b) Non-competitive and competitive detoxification of As(III) ions from single and binary biosorption systems and biosorbent regeneration. Biomass Convers Biorefinery 2023 1–28. https://doi.org/10.1007/S13399-022-03734-0

El-Sabbagh, S. M., Mira, H. I., Desouky, O. A., Hussien, S. S., Elgohary, D. M., Ali, A. O., & Naggar, A. M. A. El. (2023). Synthesis of fungal chitosan–polystyrene modified by nanoparticles of binary metals for the removal of heavy metals from waste aqueous media. RSC Advances, 13(42), 29735–29748. https://doi.org/10.1039/D3RA04451C

Kang, H. J., & Kim, J. H. (2019). Adsorption Kinetics, Mechanism, Isotherm, and Thermodynamic Analysis of Paclitaxel from Extracts of Taxus chinensis Cell Cultures onto Sylopute. Biotechnology and Bioprocess Engineering, 24(3), 513–521.

Kim, Y. S., & Kim, J. H. (2019). Isotherm, kinetic and thermodynamic studies on the adsorption of paclitaxel onto Sylopute. The Journal of Chemical Thermodynamics, 130, 104–113.

**Table S2:** Uranium desorption from loaded PBC-Zn using different solutions.

| **Eluent solution** | **Efficiency, %** |
| --- | --- |
| 1 M H_2_SO_4_ | 66 |
| 1 M HNO_3_ | 40 |
| 1 M HCl | 70 |
| 1 M Na_2_CO_3_ | 56 |
| 1M CH3COONa | 92 |

**Table S3:** Adsorption and desorption cycles for uranium recovery.

| **Recycling Investigation** | | |
| --- | --- | --- |
| **Cycle No.** | **Sorption** | **Desorption** |
| 1 | 92.6 | 91.4 |
| 2 | 91.4 | 90.8 |
| 3 | 91.1 | 90.6 |
| 4 | 89.7 | 89.2 |
| 5 | 89.6 | 88.6 |
